# Detecting *Lactococcus lactis* Prophages by Mitomycin C-Mediated Induction Coupled to Flow Cytometry Analysis

**DOI:** 10.3389/fmicb.2017.01343

**Published:** 2017-07-19

**Authors:** Joana Oliveira, Jennifer Mahony, Laurens Hanemaaijer, Thijs R. H. M. Kouwen, Horst Neve, John MacSharry, Douwe van Sinderen

**Affiliations:** ^1^School of Microbiology, University College Cork Cork, Ireland; ^2^APC Microbiome Institute, University College Cork Cork, Ireland; ^3^DSM Biotechnology Center Delft, Netherlands; ^4^Max Rubner-Institut Kiel, Germany

**Keywords:** temperate phages, lysogeny, chemical inductions, prophage, flow cytometry

## Abstract

Most analyzed *Lactococcus lactis* strains are predicted to harbor one or more prophage genomes within their chromosome; however, the true extent of the inducibility and functionality of such prophages cannot easily be deduced from sequence analysis alone. Chemical treatment of lysogenic strains with Mitomycin C is known to cause induction of temperate phages, though it is not always easy to clearly identify a lysogenic strain or to measure the number of released phage particles. Here, we report the application of flow cytometry as a reliable tool for the detection and enumeration of released lactococcal prophages using the green dye SYTO-9.

## Introduction

*Lactococcus lactis* is a non-pathogenic Gram-positive lactic acid bacterium (LAB), which is used as a starter culture for the manufacture of a variety of fermented dairy products (Rousseau and Moineau, 2009; Mahony and van Sinderen, 2014). Members of the two recognized *L. lactis* subspecies, subsp. *lactis* and subsp. *cremoris*, each impart organoleptic properties that contribute to flavor and textural characteristics of the final fermented product (Fernandez et al., 2011). Inconsistencies in the dairy production process are frequently due to (bacterio)phage infection of the lactococcal starter culture(s), which has prompted many detailed scientific studies on such bacterial viruses (Chopin et al., 2001; Marco et al., 2012). Currently, ten genetically distinct groups of lactococcal phages are known to exist, all being members of the *Caudovirales* order. The majority of these lactococcal phage groups exhibit typical characteristics of *Siphoviridae* (representing phages that possess a long, non-contractile tail), while two groups are classified as *Podoviridae* (i.e., phages possessing a short tail) (Deveau et al., 2006). Despite the apparent diversity of lactococcal phages, three groups are most commonly isolated in the environment of commercial dairy fermentations: the virulent 936 and c2 groups, and phages belonging to the P335 group whose members may be virulent or temperate (Chopin et al., 2001; Deveau et al., 2006). Lysogenic lactococcal phages that have been identified and/or characterized to date have, by and large, been assigned to the P335 group and their presence as a prophage in a lactococcal chromosome may either be considered beneficial or undesirable. In the integrated state, prophages may provide superinfection exclusion and homo-immunity against (super)infecting phages, thereby protecting their lysogenic host (McGrath et al., 2002; Labrie et al., 2010). In contrast, the threat of prophage induction and consequent cell lysis, or prophage conversion to a strictly lytic derivative constitutes a realistic risk factor to the fermentation industry (Marco et al., 2012).

The plaque assay is the standard technique used for the detection and quantification of infectious phage particles, although it is a labor-intensive and time-consuming technique, detecting infectious and virulent particles within the overall phage population (Anderson et al., 2011). Genetic techniques, in particular PCR, in combination with mitomycin C (MmC)-mediated induction growth profiles, have been described as a useful approach to identify lysogenic strains (Martín et al., 2006), where integrase-specific primers (O’Sullivan et al., 2000), and oligonucleotide primers previously described for the lactococcal P335 group detection (Labrie and Moineau, 2000) have been applied. This approach has had mixed success, as PCR-based detection only indicates the presence of a target phage sequence, but not necessarily a functional prophage, while false positive results were also reported (Martín et al., 2006). Furthermore, MmC-mediated induction growth curves do not appear to represent a very reliable procedure to detect lysogenic strains (Martín et al., 2006). In recent years, alternative techniques have been described for the purpose of virus particle detection and enumeration, including real-time PCR, the so-called nanoparticle tracking analysis (NTA)-based approach using NanoSight (NS) technology, transmission electron microscopy (TEM), fluorescence-staining methods, e.g., epifluorescence microscopy (EFM), and flow cytometry (FCM) (Marie et al., 1999; Chen et al., 2001; Anderson et al., 2011). The two former techniques provide reproducible data which are consistent with those obtained by plaque assays, though with a reduced turnaround time. Furthermore, TEM, EFM, and FCM have been assessed in detail to determine their usefulness in detecting and enumerating phage particles, revealing that EFM- and TEM-based counts often underestimate the actual virion number, whereas FCM was demonstrated to be a sensitive, (relatively) rapid and reproducible detection technique (Marie et al., 1999; Chen et al., 2001). As mentioned above, plaque assays represent a traditional methodology to detect the ability of a single phage particle to infect a permissive bacterial host, thus resulting in progeny formation (Anderson et al., 2011). However, in situations where a sensitive host may not be available for a phage, such as in the case of an induced (pro)phage, it is important to know if and how many viral particles are present (Marco et al., 2012). In the current study we report on the use of FCM as a reliable detection and enumeration method for phage particles that are released from lysogenic *L. lactis* strains following MmC treatment.

## Materials and Methods

### Bacterial Growth and Prophage Induction Conditions

*Lactococcus lactis* strains (*L. lactis* strain details are described in **Table 1** and Supplementary Table S1) were grown at 30°C in M17 broth (Oxoid) supplemented with 0.5% glucose (GM17) and prophages were chemically induced using 1.3 or 3 μg.ml^-1^ of MmC (Sigma). Four well studied laboratory *L. lactis* strains were employed in this study, i.e., *L. lactis* 3107 (Braun et al., 1989) and SMQ-86 (Emond et al., 1997), as potential sensitive hosts for lytic propagation of (some of the) temperate phages identified in this study (phage-host survey described in Supplementary Table S2), and *L. lactis* NZ9000-TP901-1*erm* (Koch et al., 1997; Stockdale et al., 2013) and UC509.9 (Costello, 1988), which were used as a positive and negative controls for flow cytometric analysis, respectively (**Table 1**). For phage propagations, 0.01 M CaCl_2_ was added to the growth medium. Phage particles were resuspended in TBT buffer (0.05 M Tris HCl [pH 7.0]; 0.1 M NaCl; 0.01 M MgCl_2_.6H_2_0) for phage DNA extraction, phage grouping by multiplex PCR, plaque assays or FCM analysis.

**Table 1:** Shortlist of lactococcal strains and phages used in this study (for a description of all lactococcal strains used see Supplementary Table S1).

Bacterial strains and phages	Relevant features	Reference
***L. lactis* strains**		
UC509.9	Prophage-free lactococcal strain (used as negative control)	Costello, 1988
NZ9000-TP901-1*erm*	Strain carrying the inducible prophage TP901-1*erm* (used as positive control)	Koch et al., 1997; Stockdale et al., 2013
SMQ-86	Sensitive host strain for phage-host survey and propagation of induced (pro)phage	Emond et al., 1997
3107	Sensitive host strain for phage-host survey and propagation of induced (pro)phage	Braun et al., 1989
**(Pro)phages**		
TP901-1*erm*	Inducible temperate phage, used as a positive control	Koch et al., 1997; Stockdale et al., 2013
56701	Temperate phage induced from *L. lactis* DS68567	Oliveira et al., 2016
98201	Temperate phage induced from *L. lactis* DS64982	Oliveira et al., 2016
28201	Temperate phage induced from *L. lactis* DS70282	Oliveira et al., 2016
50901	Temperate phage induced from *L. lactis* DS68509	Oliveira et al., 2016
62501	Temperate phage induced from *L. lactis* DS63625	Oliveira et al., 2016
18301	Temperate phage induced from *L. lactis* DS72183	This study
58501	Temperate phage induced from *L. lactis* DS68585	This study
38501	Temperate phage induced from *L. lactis* DS70385	This study
16001	Temperate phage induced from *L. lactis* DS72160	This study
15901	Temperate phage induced from *L. lactis* DS72159	This study
07501	Temperate phage induced from *L. lactis* DS69075	This study
63301	Temperate phage induced from *L. lactis* DS63633	Oliveira et al., 2016
50101	Temperate phage induced from *L. lactis* DS68501	Oliveira et al., 2016
58601	Temperate phage induced from *L. lactis* DS68586	This study
86501	Temperate phage induced from *L. lactis* DS71865	Oliveira et al., 2016
24801	Temperate phage induced from *L. lactis* DS70248	This study
06701	Temperate phage induced from *L. lactis* DS69067	This study
49801	Temperate phage induced from *L. lactis* DS68498	This study
51801	Temperate phage induced from *L. lactis* DS68518	This study
49501	Temperate phage induced from *L. lactis* DS68495	This study


### Small and Large Scale Prophage Inductions

Small-scale prophage induction trials were performed in 96-well microtitre plates by (in each well) inoculating 200 μl of GM17 broth with 2% fresh overnight culture of a particular *L. lactis* strain. Chemical induction was performed in early exponential phase (OD_600nm_∼0.2) by the addition of MmC at a final concentration of 0 (i.e., without MmC; negative control), 1.3 or 3 μg.ml^-1^. Incubation was continued at 30°C and bacterial growth was followed for 8 h. A final OD_600nm_ reading was obtained 24 h after the lactococcal strains were inoculated in the 96-well microplate, and growth profiles were then generated. In order to obtain cell lysates, MmC-mediated inductions were performed as described before, but at a large scale. Briefly, this involved the addition of 3 μg.ml^-1^ of MmC (final concentration) to early exponential phase cultures grown in a 50 ml volume of GM17 broth, followed by overnight incubation at room temperature. Cell debris was removed by centrifugation at 7560 × *g* for 20 min and lysates were then passaged through a 0.45 μm filter (Sarstedt, Nümbrecht Germany).

### Phage Propagation and Isolation

To assess if prophage induction resulted in the production of infective phage particles, lysates (see previous section) were employed for a phage susceptibility analysis, involving two strains previously described as a sensitive host to several P335 phages, i.e., *L. lactis* 3107 and SMQ-86. This phage-host range analysis was performed by plaque assays as previously described (Lillehaug, 1997). Where a host was identified for a (MmC-induced) phage, propagations using single plaques were performed to ensure that a pure phage with a single genotype was propagated, while serial propagations in GM17 broth were performed in order to increase the phage titer. A final propagation of temperate phage(s) was performed using a 1% inoculum of the bacterial host in 50 ml of broth supplemented with 0.01 M CaCl_2_ (final concentration) and to which 1% (v/v) of phage lysate with a titer between 10^8^ and 10^9^ plaque-forming units per ml (pfu.ml^-1^) was added. The phage-host mixture was incubated at 30°C until visible lysis was observed, after which remaining cells were removed from the lysate by passage through a 0.45 μm filter, followed by storage at 4°C.

### Phage DNA Extraction and Multiplex PCR

Filtered phage lysates were treated with DNase and RNase to remove residual host chromosomal DNA and RNA, and incubated at 37°C for 40 min before adding polyethylene glycol (PEG_8000_) to a final concentration of 10% w/v, followed by incubation at 4°C for 16 h. Phage DNA extraction was performed as described previously (Mahony et al., 2013), with minor modifications (Moineau et al., 1994). Phage genotyping was performed by a previously established multiplex PCR methodology (Labrie and Moineau, 2000), in which three different primer pairs, each based on group-specific regions of the three dominant lactococcal phage groups (i.e., the 936, P335 and c2 groups) were employed. Phage DNA or phage lysate was used as a template, and the PCR products were generated as previously described (Labrie and Moineau, 2000). PCR products were separated by gel electrophoresis on a 1% agarose gel and visualized by UV illumination.

### Detection of Prophages by Flow Cytometry

Following large scale induction, as described above, released phage particles were detected by FCM using the LIVE/DEAD BacLight bacterial viability and counting kit (Life Technologies). Briefly, bacterial debris were removed by centrifugation (9148 × *g* for 20 min) following addition of 0.5 M NaCl (final concentration) and subsequent incubation for 2 h at 4°C with agitation. Phage particles were precipitated by the addition of 10% PEG_8000_ to filtered cell-free supernatant followed by overnight incubation at 4°C and subsequent recovery by centrifugation at 17,620 × *g* for 15 min. The resulting (virion-containing) pellet was resuspended in 1 ml TBT buffer followed by two washes (10,000 × *g*) in 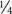 strength Ringer’s solution as described in the LIVE/DEAD BacLight bacterial viability and counting kit (Thermo Fisher Scientific, Leiden, The Netherlands). Following incubation for 30–60 min at room temperature, a final wash step was performed and the pellet was then resuspended in 1 ml of 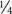 strength Ringer’s solution. A 1:10 dilution of this phage suspension was prepared in 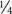 strength Ringer’s solution, stained with 0.15% of the SYTO-9 nucleic acid dye (light protected) and analyzed by FCM in triplicate. Of note, the same phage suspension prepared in 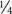 strength Ringer’s solution was additionally used for plaque assays analysis, in order to see the reliability of the FCM in phage enumeration. FCM analysis was performed using the BD Accuri C6 flow cytometer by detection of excitation/emission wavelengths from SYTO-9–stained DNA (485/498 nm, respectively). Briefly, measurements were performed in logarithmic scale based on the following parameters: run limits for 5000 events.ml^-1^; medium flow rate (33 μl.min^-1^) and a threshold set on forward scatter (FSC-A) to allow for the discrimination of phages (from background noise; see Supplementary Figure S1). Phage particle quantification was obtained by applying the following formula: [(no. events detected/sample volume analyzed)^∗^10] (pfu.ml^-1^) for all lactococcal strains used. In order to obtain an accurate phage particle quantitation by FCM, each analysis was conducted to acquire an equal amount of events per sample (5000 events.ml^-1^) and/or in instances where samples presented with low particle numbers, the same sampling time was maintained to ensure equal sampling. Positive (phage TP901-1 induced from NZ9000-TP901-1) (Koch et al., 1997; Stockdale et al., 2013) and negative (*L. lactis* strain UC509.9, which is prophage-free) (Costello, 1988) controls were also included in the FCM analysis. SYTO-9 emissions were detected in the FL-1 channel (BP Filter 530/30), and data analysis was performed using FCS 5 Express plus software (described in the Supplementary Figure S1).

### Temperate Phage Particle Detection by Transmission Electron Microscopy (TEM)

For TEM analysis phage particles produced from MmC-treated cultures (in G-M17 growth broth) were adsorbed to a carbon-coated 400-mesh copper grid (Agar Scientific, Essex, United Kingdom) and negative staining with 2% (w/v) uranyl acetate was performed as described previously (Deasy et al., 2011). Specimens were examined with a Tecnai 10 transmission electron microscope (FEI Thermo Fisher Scientific, Eindhoven, The Netherlands) operated at an acceleration voltage of 80 kV. Micrographs were taken with a MegaView G2 charge-coupled-device camera (EMSIS, Münster, Germany). All measurements of the phage particle dimensions were performed using iTEM imaging software (EMSIS).

## Results

### Small Scale Prophage Induction Profile Analysis

**FIGURE 1:**
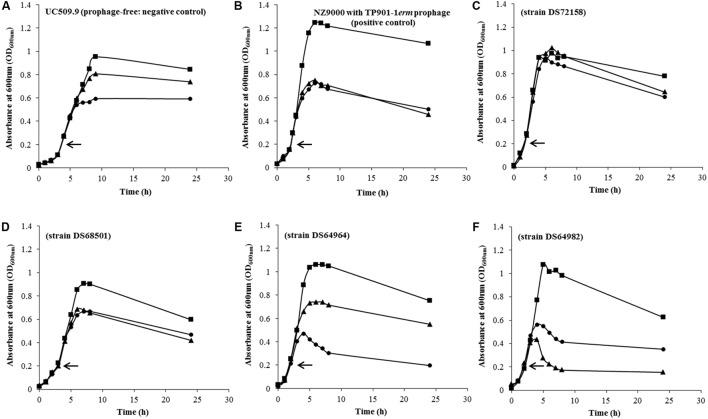
Representative growth curves profiles observed in two *L. lactis* control strains and 113 *L. lactis* strains used in this study during a chemical induction (MmC) at different concentrations: 0 μg.ml^-1^ (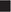); 1.3 μg.ml^-1^ (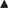) and 3 μg.ml^-1^ (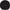). Chemical induction was performed in early exponential phase (OD_600nm_∼0.2) as indicated (←). **(A)** *L. lactis* UC509.9 (negative control); **(B)** *L. lactis* NZ9000 containing the inducible TP901-1*erm* phage (positive control); **(C)** *L. lactis* DS72158 strain as a representation of strains considered to be non-inducible (and thus by inference prophage-negative) strain in the presence of 1.3 and 3 μg.ml^-1^ of MmC; **(D)** *L. lactis* strain DS68501 as a representative strain where addition of MmC at either low or high concentrations caused an equal drastic impact on growth; **(E)** *L. lactis* strain DS64964 as a representative of *L. lactis* strains exhibiting a complete cessation of growth and partial lysis upon addition of the highest concentration MmC (3 μg.ml^-1^); **(F)** *L. lactis* strain DS64982 as a representative strain where addition of the lower of the two tested MmC concentrations (1.3 μg.ml^-1^) was shown to elicit a significant drop in OD value, being more pronounced than that shown for the higher MmC concentration.

113 *L. lactis* strains were assessed for the presence of inducible prophages following treatment with two different MmC concentrations. Optical density of treated cultures was monitored over a period of 24 h at 30°C and compared to a corresponding untreated control culture (i.e., same strain but without MmC addition; **Figure 1**). Growth profiles were obtained and compared with growth profiles from two lactococcal strains used as a negative or positive control: *L. lactis* UC509.9, which is free of inducible prophages (Costello, 1988), and *L. lactis* NZ9000-TP901-1*erm*, which contains an inducible prophage (Koch et al., 1997; Stockdale et al., 2013), respectively. Following addition of 1.3 or 3 μg.ml^-1^ of MmC, the observed growth profiles for the positive control revealed a drastic growth reduction of the strain when exposed to MmC (as compared to untreated control), with an equal impact observed for either of the two MmC concentrations (**Figure 1B**; this growth behavior is referred to as growth profile B). In contrast, the negative control {*L. lactis* UC509.9 [a prophage-free strain] (Costello, 1988)} was shown to exhibit a gradual impact on growth when exposed to MmC, with a more pronounced diminishment of growth at the higher MmC concentration, possibly due to the toxic effect of MmC (**Figure 1A**; this growth behavior is referred to as growth profile A). To try to correlate cell lysis with prophage induction, growth profile comparisons between 113 tested lactococcal strains and control strains was performed (**Figure 1** and Supplementary Table S1). Interestingly, in addition to the two growth profiles A and B as identified above, two additional and distinct MmC-mediated growth profiles were observed.

Detailed assessment of the growth profiles obtained from this lactococcal strain collection showed that the growth behavior of 33.63% of the lactococcal strains screened was reminiscent of growth profile B as observed for the positive control (NZ9000-TP901-1*erm*; similar profiles were observed in **Figures 1B,D**), suggesting that these strains may harbor one or more inducible prophages. Furthermore, the growth behavior of 36.28% of the lactococcal strain screened was similar to growth profile A, i.e., the negative control used in this study (UC509.9; similar profiles were observed in **Figures 1A,E**). Interestingly, 15.93% of *L. lactis* strains tested in this study displayed similar growth profiles to the corresponding untreated strain (**Figure 1C**), i.e., no significant growth arrest was observed following MmC addition (compared to growth in the absence of MmC), and this was taken as an indication that no prophage induction had taken place (this behavior is referred to as growth profile C). Finally, the remaining 14.16% of the tested lactococcal strains exhibited a more severe growth arrest (and substantial cell lysis) in the presence of 1.3 μg.ml^-1^ MmC compared to that observed for the same culture following exposure to 3 μg.ml^-1^ MmC (**Figure 1F** and referred here as growth profile D). The substantial drop in bacterial growth observed following addition of 1.3 μg.ml^-1^ of MmC (and less so at the higher concentration of MmC) may be due to phage induction (and consequent cell lysis) rather than MmC toxicity (as one may expect this to cause similar or incremental growth cessation profiles).

### Characterization of Induced Temperate Phages

In order to validate the identification of putative lysogens among the 113 tested strains, chemical induction was performed on a larger scale (50 ml and addition of 3 μg.ml^-1^ MmC; see Materials and Methods). The resulting lysates were tested for the presence of infectious phages in a phage-host survey against two highly phage-sensitive *L. lactis* strains, 3107 (Braun et al., 1989) and SMQ-86 (Emond et al., 1997), in an effort to find a suitable propagation host. The results of this phage-host survey are summarized in Supplementary Table S2 with the identification of MmC-inducible phages from fifteen *L. lactis* strains capable of infecting either *L. lactis* SMQ-86 or *L. lactis* 3107. Interestingly, a third of these strains were shown to exhibit a growth profile that was similar to that of the negative control UC509.9 following MmC exposure (i.e., growth profile A), indicating that comparative growth profile analysis (following MmC treatment) is not a very dependable method to determine if a strain harbors (an) inducible prophage(s).

### Direct Detection of Temperate Phages Induced from Lysogenic Strains by Flow Cytometry Analysis

In order to assess if released phage particles (following induction from a lysogenic host) can be detected and quantified by FCM, we tested this technology by employing MmC-mediated lysates in which the DNA-binding dye SYTO-9 had been incorporated together with a calibrated suspension of microspheres (6.0 μm) to accurately estimate the volume analyzed (see Materials and Methods; Supplementary Figure S1). FCM optimization and subsequent lactococcal lysate analysis is discussed below.

#### Establishment of Flow Cytometry Procedure for Phage Particle Detection

**FIGURE 2:**
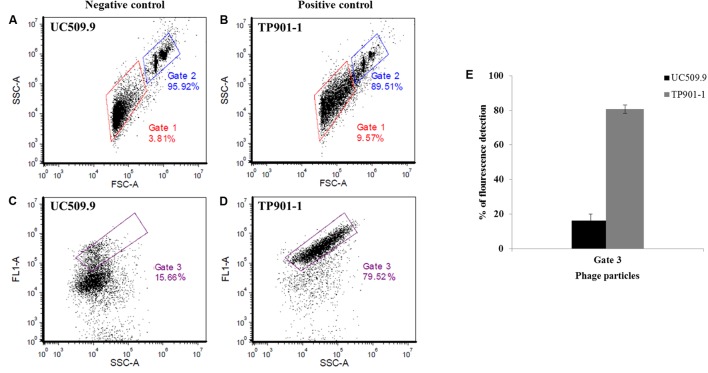
Representative cytograms for SYTO9-stained samples of two *L. lactis* controls using the BD Accuri^TM^ C6 flow cytometer. **(A,C)** Cytograms of MmC-treated *L. lactis* UC509.9 (prophage-free lactococcal strain used as negative control); **(B,D)** Cytogram of MmC-treated *L. lactis* NZ9000-TP901-1*erm* (lactococcal strain harboring the TP901-1 prophage used as a positive control); **(A,B)** Representative cytograms of the gating strategy for size discrimination between the phage-containing (induced) sample (Gate 1, red) and the control 6.0 μm microsphere suspension (Gate 2, blue), using SSC (Side Scattered light) *versus* FSC (Forward Scattered light) analysis. **(C,D)** Representative cytogram for the measurement of fluorescence detection (FL1/SYTO-9) in the phage population *versus* SSC, identified in Gate 1 in **(A,B)** mentioned above, which was subsequently selected in Gate 3 (purple). Gate 3 was applied to identify the percentage of fluorescence detected by phage DNA- containing particles stained with SYTO-9. **(E)** Percentage of SYTO-9 fluorescence detected in the Gate 3 applied in two inducible *L. lactis* strains (positive and negative control) using 3 μg.ml^-1^ of MmC.

Phage particle detection by means of FCM was first explored through the analysis of two different controls (**Figure 2**); one positive control (phage TP901-1 induced from the lysogenic strain NZ9000-TP901-1*erm*) and one negative control (UC509.9 [prophage-negative]). All data were acquired from three independent experiments, and using the FCS Express 5 Plus software^1^. The obtained MmC-mediated lysates from these two controls were analyzed by FCM through the measurement of scattered light in the forward and side scatter directions (FSC and SSC, respectively) (**Figures 2A,B** and Supplementary Figure S1). Both FSC and SSC are unique for every particle, and the combination of these two scatter data sets allows the distinction between two different particles based on size and complexity: lysate particles (virions and other particles produced by cell lysis) and microspheres, represented by Gates 1 and 2, respectively. In order to identify phage particles (potentially) present in our lysate (Gate 1), the fluorescence emitted from stained phage DNA was measured using the FL1 channel (**Figures 2C,D** and Supplementary Figure S1) and detected in Gate 3. The fluorescence levels detected in Gate 3 for the positive and negative controls show that they emit clearly distinguishable fluorescent signals (**Figure 2E** and Supplementary Figure S1). Briefly, for the employed positive control (phage TP901-1 induced from NZ9000), a high level of fluorescence was detected in the virus particle gate region (Gate 3; Supplementary Figure S1). In detail, 80.60% fluorescence was detected for phage lysates of TP901.1, thus revealing the detection of SYTO-9 stained phage particles. For the negative control strain (UC509.9) employed in this study, a low and presumed background level of fluorescence (16.33%; **Figure 2** and Supplementary Figure S1) was emitted in the viral particle gate region (Gate 3), being consistent with the absence of phage particles in this MmC-treated strain. Based on the results obtained from these *L. lactis* controls it thus appeared feasible to establish a reliable FCM analysis for phage particle detection.

#### Flow Cytometry Analysis of MmC-Induced *L. lactis* Strains

**FIGURE 3:**
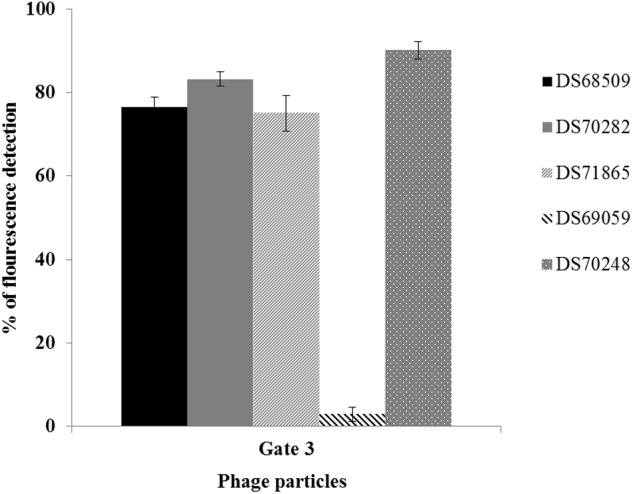
Percentage of SYTO-9 fluorescence detected in the Gate 3 applied in five inducible *L. lactis* strains using 3 μg.ml^-1^ of MmC. Gate 3 was applied to identify the percentage of fluorescence detected by phage-DNA particles stained with SYTO-9.

The protocol developed to distinguish a lysogenic from a non-lysogenic strain (as described above) was subsequently applied to detect (temperate) phages released from MmC-treated lactococcal strains (**Figure 3** and **Table 2**). FCM analysis was performed using a selection of eighteen lactococcal strains: seven strains which upon MmC treatment produce lysates containing phages for which a suitable host had been identified (**Table 3** and Supplementary Table S2); and eleven strains, which represent each of the four previoulsy described MmC-mediated growth profiles (see above; Supplementary Table S1) and for which we could not detect phages in the resulting lysate as based on a plaque assay (**Figure 3** and **Tables 2**, **3**). From **Figure 3**, a pattern of SYTO-9-stained lysates can be observed that allows the identification of bacterial strains that either do or do not release temperate phages following MmC treatment (**Table 2**). As expected, the seven *L. lactis* strains (**Table 3**), which upon MmC treatment released phages that lytically infect 3107 or SMQ-86 (see above), were all shown to emit high fluorescence signals due to the presence of DNA-stained phage particles (**Figure 3** and **Tables 2**, **3**). As mentioned above, despite the identification of a sensitive host for the phages released by these seven lysogenic strains, only some of these strains were shown to exhibit MmC-mediated growth profile similar to that of TP901-1 (*L. lactis* strains DS63633 and DS70282; **Table 3** and Supplementary Table S1). The same approach was applied to test for the presence of particles in eleven lysates obtained upon MmC treatment for which no suitable host had been identified (**Tables 2**, **3**). It was observed that the lysates obtained (following MmC exposure) from *L. lactis* strains DS64964, DS601, DS69059, DS66563, DS68569, and DS72158 were shown to be associated with a low level SYTO-9 fluorescence signal (<12.05%; **Tables 2**, **3**). This level of fluorescence is somewhat lower than that observed for the negative control strain (UC509.9; **Table 3** and Supplementary Figure S1) and it was therefore concluded that these strains do not release (DNA-containing) phage particles upon MmC treatment. Interestingly, despite their apparent inability to release phage particles, it was observed that two of these six strains exhibit a MmC-mediated growth profile similar to that observed for the UC509.9 (growth profile A; negative for phage particles release; **Table 3**) and another two exhibited a growth profile C (above described).

**Table 2:** Percentage of fluorescence detected in five inducible lysates stained with SYTO-9 dye.

*L. lactis* strains tested	Features		Flow cytometry analysis (% of fluorescence) associated with Gate 3	
		
	Induced prophage	*L. lactis* host	Virion-associated	Background
DS68509	50901	SMQ-86	76.49 ± 5.18	23.51 ± 5.18
DS70282	28201	SMQ-86	83.19 ± 4.37	16.81 ± 4.37
DS71865	86501	3107	75.03 ± 8.20	24.97 ± 8.20
DS69059	–	–	2.86 ± 0.43	97.14 ± 0.43
DS70248	24801	–	90.03 ± 5.21	9.97 ± 5.21


**Table 3:** Phage particles detection in inducible *L. lactis* lysates and correlation between flow cytometry and MmC growth profiles.

*L. lactis* strains	MmC growth profile	Inducible prophage	*L. lactis* host	Plaque assays (pfu.ml^-1^)	Phage-group^1^	Flow cytometry analysis (pfu.ml^-1^)^∗^	Gate 3 (% fluorescence)	Electron microscopy^#^
**Controls**								
NZ9000 (TP901-1 erm)	B	TP901-1	3107	2.43e05	P335	6.85e05	80.6	nd
UC509.9	A	Prophage-free	–	–	–	<1.0e04	16.3	nd
**Strains tested**								
DS68567	A	56701	SMQ-86	5.90e04	P335	1.74e06	48.6	nd
DS71865	A	86501	3107	8.53e03	P335	9.12e05	75.0	+
DS63633	B	63301	3107	2.07e05	P335	1.79e05	29.7	+
DS70282	B	28201	SMQ-86	8.30e03	P335	1.59e06	83.2	nd
DS63625	D	62501	SMQ-86	2.07e04	P335	5.47e05	72.7	nd
DS68509	D	50901	SMQ-86	8.88e04	P335	1.24e06	76.5	+
DS64982	D	98201	SMQ-86	6.55e04	P335	1.12e06	55.9	+
DS64964	A	No prophage detected	–	–	nd	<1.0e04	0.37	nd
DS601	A	No prophage detected	–	–	nd	<1.0e04	2.92	–
DS69067	A	06701	–	–	nd	2.07e07	99.79	+
DS68498	B	49801	–	–	nd	5.22e06	99.13	+
DS69059	B	No prophage detected	–	–	nd	<1.0e04	2.86	nd
DS68518	B	51801	–	–	nd	2.02e06	97.48	nd
DS66563	B	No prophage detected	–	–	nd	<1.0e04	5.28	–
DS68569	C	No prophage detected	–	–	nd	<1.0e04	11.14	nd
DS72158	C	No prophage detected	–	–	nd	<1.0e04	12.05	–
DS70248	D	24801	–	–	nd	3.80e06	90.03	+
DS68495	D	49501	–	–	nd	4.04e06	98.37	nd


***Profile A:** UC509.9 profile with a growth cessation at 3 μg.ml^-*1*^; **Profile B:** TP901-1 profile with an equal growth cessation (1.3 and 3 μg.ml^-*1*^); **Profile C:** No chemical effect; **Profile D:** Growth cessation at 1.3 μg.ml^-*1*^; nd, Not determined; ^*1*^ Phage-group was determined by Multiplex PCR; ^∗^Phage particles quantification was performed using the following equation: [(no. events detected/sample volume analyzed)^∗^10] (pfu.ml^-*1*^). ^#^+: Presence of phage particles; ^#^-: phage particles not detected.*

The lysates of the remaining five strains (i.e., five out of the eleven with no suitable sensitive host identified), i.e., DS69067, DS68498, DS68518, DS70248, and DS68495, were shown to exhibit high fluorescence signals (>90%), clearly indicative of phage particle release. Notably, only two of these strains (DS68498 and DS68518) were shown to exhibit a MmC-mediated growth profile related to that observed for the positive control (growth profile B), while two other strains were shown to exhibit growth profile D (described above).

### TEM Analysis of Temperate Lactococcal Phages

**FIGURE 4:**
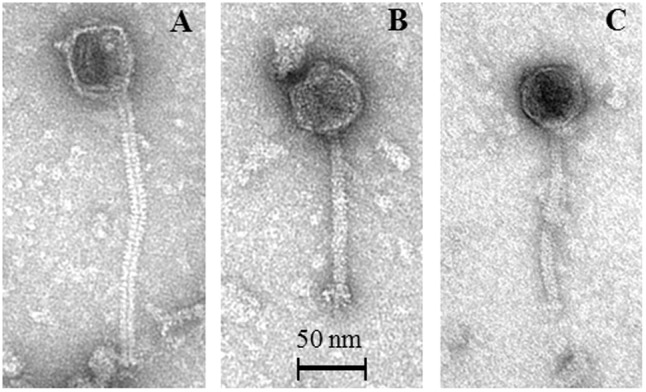
Representative transmission electron micrographs of P335-like phages released from MmC-treated lactococcal strains. **(A)** Phage 06701 released from *L. lactis* strain DS69067; **(B)** Phage 24801 released from *L. lactis* strain DS70248; **(C)** Phage 49801 released from *L. lactis* strain DS68498.

In order to substantiate the notion that FCM can reliably detect phage particles induced from lysogenic strains (in particular when no suitable sensitive host is available for such phages), TEM analysis was performed on MmC-mediated lysates of six strains, which were selected based on their distinct MmC-mediated growth profile and FCM results (DS601, DS69067, DS68498, DS66563, DS72158, and DS70248; **Table 3**). This TEM analysis revealed that the MmC-mediated lysates of three strains (i.e., DS69067, DS68498, and DS70248) harbored phage particles (**Figure 4**). Of these three strains, only DS68498 was shown to exhibit a MmC-mediated growth profile similar to that of the positive control. Analysis of the images revealed that all phages possess an isometric capsid and a non-contractile tail measuring with approximately 57–60 nm and 117–190 nm (**Figure 4**), in some cases complex baseplates were also observed (**Figure 4B**), similar to that noted for other members of the P335 phage group (Labrie et al., 2008; Mahony et al., 2017). In contrast, no observable phage particles were detected in the lysates of strains DS601, DS66563, and DS72158 by TEM with a limit of detection of 10^5^ to 10^6^ particles per ml (Zhang et al., 2013). Based on our data it is clear that MmC-mediated growth profile analysis is not a reliable method to identify lysogenic strains; in contrast, the results obtained from FCM and TEM analyses are fully consistent with each other, suggesting that the former is a reliable and relatively rapid method to establish if a strain harbors inducible prophages.

### Phage Detection Using Flow Cytometry: Plaque Assays versus FCM Total Virus Validation

**FIGURE 5:**
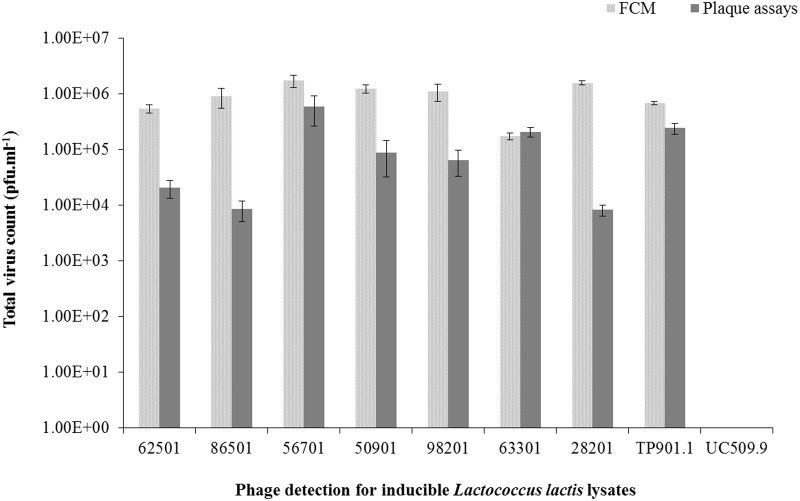
Comparative analysis between flow cytometry and plaque assay techniques for the detection of total virus particles (pfu.ml^-1^) in seven *L. lactis* strains, positive and negative controls induced using 3 μg.ml^-1^ of MmC.

We also wanted to validate FCM as a reliable method for the quantitation of viral particles (in particular when a suitable host is unavailable to perform plaque assays). For this purpose, using the 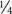 strength Ringer’s phage suspension following PEG precipitation of MmC-mediated lysates, phage particle quantification values obtained from FCM (see Material and Methods) and plaque assays were compared (**Figure 5**) for seven strains (out of fifteen lysates, for which a sensitive host strain had been identified by phage-host survey; Supplementary Table S2). Plaque assays on the inducible prophages were performed using two lactococcal hosts: *L. lactis* subsp. *lactis* SMQ-86 or *L. lactis* subsp. *cremoris* 3107 (**Figure 5** and Supplementary Table S2). The phage numbers obtained by plaque assays and FCM were compared, and *L. lactis* UC509.9 (phage-negative sample) was used to normalize the total phage count from FCM analysis and additionally, to estimate the limit of phage particle detection by FCM. Based on our comparisons, lactococcal lysates (following MmC treatment) that by FCM analysis exhibit fluorescence signals below 16% and a phage particle number of less than 10^4^ pfu.ml^-1^ are considered to be negative for phage particles (**Table 3**). While FCM is unable to distinguish between infectious and non-infectious viral particles (Bosch et al., 2004), the number of phage particles detected by FCM was in most cases found to be in the same order of magnitude as the titre obtained from the plaque assay method (where this was possible). In cases where a more pronounced difference was observed [i.e., where FCM data indicates a more than 10-fold higher number of phage particles; e.g., *L. lactis* strains harboring prophages DS64982, DS71865, DS70282, and DS63625 (**Figure 5**)], this difference may be explained by the possibility that not all phage particles present are infectious (confirmed by detection of disintegrated particles and empty capsids by TEM, data not shown) or that the host encodes a phage-resistance system that reduces the efficacy of plaquing.

## Discussion

The current study assessed if FCM is a suitable method to detect virus particles induced from lysogenic *L. lactis* strains. Several studies have indicated that the majority of lactococcal strains harbor one or more prophage-like sequences in their genome (Chopin et al., 2001; Ventura et al., 2007; Mahony et al., 2008). MmC, UV and temperature treatments are among the most commonly reported methods to achieve prophage induction (Chopin et al., 2001; Nanda et al., 2015; Ho et al., 2016). As reported here, MmC-mediated growth profile analysis is of limited value due to many false positive/negative results thereby highlighting the need for an accurate phage particle detection and enumeration method (Martín et al., 2006). We show here that FCM can be employed as a reliable method for the identification of lysogenic bacteria and detection of released phage particles. Marie and co-workers first described an approach in 1999 (Marie et al., 1999) to detect marine viruses by FCM based on viral infection of *Phaeocystis pouchetii*. Through analysis of uninfected host bacteria these authors showed that it was possible to differentiate between background signal and viral particles (Marie et al., 1999). In our study, using FCM it was possible to reliably detect and enumerate phage particles, although it is not possible to determine if such particles represent one or multiple distinct phages. Validation of FCM as a reliable method for the detection and quantitation of viral particles was achieved using (i) plaque assays (in cases where a suitable host was available to perform plaque assays), and (ii) TEM (where no suitable host was available to perform plaque assays). Despite the fact that the double-agar plaque assay is probably the most efficient method for intact (i.e., infectious) phage detection and quantification, this approach cannot be employed in situations where a suitable host cannot be identified for a released (pro)phage. Lambeth et al. (2005) demonstrated the application of FCM-based assays for dengue virus particle quantifications as a useful technique to titrate clinical isolates of dengue that frequently do not form clear plaques (Lambeth et al., 2005). Several techniques have been applied to detect (induced) temperate phages, such as real time PCR (Lunde et al., 2003), bacterial genome sequencing analysis (Chopin et al., 2001), TEM and EFM analysis (Marie et al., 1999; Chen et al., 2001). FCM has previously been applied for real-time detection of *L. lactis* infected with c2-type phages in order to detect the early stages of infection, aimed at improving the management of dairy fermentation processes (Michelsen et al., 2007). In contrast to our approach for phage particle detection, the latter study was intended to differentiate between phage-infected and uninfected lactococcal populations.

Interestingly, our results obtained from FCM and TEM analyses are fully consistent with each other, where it was possible to identify intact phage particles with morphological features typical of the P335 phages (Mc Grath et al., 2006; Veesler et al., 2012; Mahony et al., 2017). Despite the fact that TEM analysis is a very useful means by which to visualize virus particles, this technique comes with certain limitations such as lengthy sample preparation procedures, expensive equipment and specialized expertise (Goldsmith and Miller, 2009; Vale et al., 2010; Zhang et al., 2013; Brown et al., 2015). Thus, only a relatively small number of strains were subjected to TEM analysis. FCM has shown to be a relatively fast and accurate tool for the identification of inducible phage particles when a sensitive host cannot be applied, reducing the need for TEM access. FCM is a rapid technique to perform an analysis of multiple parameters of individual cells and with important applications in food microbiology (Comas-Riu and Rius, 2009; Paparella et al., 2012). Prevention of phage infection has received a lot of attention in recent years, and several strategies have been employed such as the application of rotating cultures and phage-resistant starter strains (Michelsen et al., 2007). The FCM technique described here will assist in reliable identification of potential problematic starter cultures, and will thus ultimately reduce the risk of phage particles being released in the dairy environment.

## Author Contributions

JO wrote the manuscript. JO, JM, and DvS were involved in the idea conception and manuscript editing. JO, LH, HN, and JMS were involved in the experimental design and/or data analysis. JM, TK, and DvS were involved in manuscript editing.

## Conflict of Interest Statement

JO is funded by DSM Food Specialties and LH and TK are employees of DSM Food Specialties. The other authors declare that the research was conducted in the absence of any commercial or financial relationships that could be construed as a potential conflict of interest.

## Footnotes

1 www.denovosoftware.com
