# Supplementary material for: Detecting Lactococcus lactis Prophages by Mitomycin C-Mediated Induction Coupled to Flow Cytometry Analysis
Source: Front Microbiol. 2017 Jul 19;8:1343. doi: 10.3389/fmicb.2017.01343 (PMC5515857; doi:10.3389/fmicb.2017.01343)
Supplement: Supplementary file 1 [file Table_1.PDF]

Table S1. General features of the *L. lactis* strains used in this study.

***L. lactis* strains**	***L. lactis* subsp.**	**MmC growth profile**	**Phage particle detection**			
			**Flow cytometry#**	**Electron microscopy#**	**Plaque assays#**	**Host***
DS72152	*cremoris*	C	nd	nd	nd	nd
DS72153	*cremoris*	A	nd	nd	nd	nd
DS65024	*cremoris*	B	nd	nd	nd	nd
DS72154	*cremoris*	B	nd	nd	nd	nd
DS72155	*cremoris*	C	nd	nd	nd	nd
DS72156	*cremoris*	C	nd	nd	nd	nd
DS68515	*cremoris*	B	nd	nd	nd	nd
DS68490	*cremoris*	A	nd	nd	nd	nd
DS65655	*cremoris*	A	nd	nd	nd	nd
DS68491	*cremoris*	A	nd	nd	nd	nd
DS68492	*cremoris*	B	nd	nd	nd	nd
DS72157	*cremoris*	A	nd	nd	nd	nd
DS68493	*cremoris*	B	nd	nd	nd	nd
DS68517	*cremoris*	B	nd	nd	nd	nd
DS68494	*cremoris*	B	nd	nd	nd	nd
DS68518	*lactis*	B	+	nd	nd	nd
DS68495	*cremoris*	D	+	nd	nd	nd
DS68496	*lactis*	D	nd	nd	nd	nd
DS68567	*cremoris*	A	+	nd	+	+
DS68568	*cremoris*	C	nd	nd	nd	nd
DS68569	*cremoris*	C	-	nd	nd	nd
DS68498	*cremoris*	B	+	+	nd	nd
DS68499	*cremoris*	B	nd	nd	nd	nd
DS68500	*cremoris*	A	nd	nd	nd	nd
DS68501	*cremoris*	B	+	+	nd	+
DS72163	*lactis*	C	nd	nd	nd	nd
DS72164	*cremoris*	B	nd	nd	nd	nd
DS68507	*lactis*	B	nd	nd	nd	nd
DS68570	*cremoris*	C	nd	nd	nd	nd
DS68571	*lactis*	C	nd	nd	nd	nd
DS68572	*cremoris*	B	nd	nd	nd	nd
DS71860	*cremoris*	B	nd	nd	nd	nd
DS68574	*cremoris*	C	nd	nd	nd	nd
DS68578	*cremoris*	D	nd	nd	nd	nd
DS68579	*cremoris*	A	nd	nd	nd	nd
DS72166	*cremoris*	D	nd	nd	nd	nd

Profile A: UC509.9 profile with a growth cessation at 3 µg.ml^{-1}; **Profile B:** TP901-1 profile with an equal growth cessation (1.3 and 3 µg.ml^{-1}); **Profile C:** No chemical effect; **Profile D:** Growth cessation at 1.3 µg.ml^{-1}; For the inducible lysates: **nd-** Not determined; # +: presence of phage particles; # -: phage particles not detected; * +: lytic host identified; * -: no lytic host.

Table S1. (cont.)

L. lactis strains	*L. lactis* subsp.	MmC growth profile	Phage particle detection			
			Flow cytometry#	Electron microscopy#	Plaque assays#	Host*
DS68509	*lactis*	D	+	+	+	+
DS68581	*cremoris*	B	nd	nd	nd	nd
DS68583	*cremoris*	A	nd	nd	nd	nd
DS68584	*cremoris*	C	nd	nd	nd	nd
DS71869	*cremoris*	A	nd	nd	nd	nd
DS69075	*cremoris*	A	nd	nd	nd	+
DS69076	*cremoris*	A	nd	nd	nd	nd
DS69077	*cremoris*	C	nd	nd	nd	nd
DS69078	*cremoris*	B	nd	nd	nd	nd
DS69080	*cremoris*	C	nd	nd	nd	nd
DS72158	*cremoris*	C	-	-	nd	nd
DS71861	*cremoris*	A	nd	nd	nd	nd
DS69081	*cremoris*	C	nd	nd	nd	nd
DS69082	*cremoris*	C	nd	nd	nd	nd
DS72167	*cremoris*	A	nd	nd	nd	nd
DS72183	*lactis*	D	nd	nd	nd	+
DS72159	*lactis*	D	nd	nd	nd	+
DS68585	*lactis*	B	nd	nd	nd	+
DS72160	*cremoris*	A	+	nd	nd	+
DS68586	*cremoris*	A	nd	nd	nd	+
DS71865	*cremoris*	A	+	+	+	+
DS71867	*cremoris*	A	nd	nd	nd	nd
DS71868	*cremoris*	A	nd	nd	nd	nd
DS71864	*cremoris*	A	nd	nd	nd	nd
DS72168	*cremoris*	B	nd	nd	nd	nd
DS71863	*cremoris*	A	nd	nd	nd	nd
DS70756	*cremoris*	D	nd	nd	nd	nd
DS70757	*cremoris*	A	nd	nd	nd	nd
DS70758	*cremoris*	A	nd	nd	nd	nd
DS70759	*cremoris*	C	nd	nd	nd	nd
DS70760	*cremoris*	C	nd	nd	nd	nd
DS70761	*cremoris*	C	nd	nd	nd	nd
DS70762	*cremoris*	A	nd	nd	nd	nd
107A	*lactis*	A	nd	nd	nd	nd
107B	*lactis*	D	nd	nd	nd	nd
107C	*lactis*	B	nd	nd	nd	nd
DS68040	*cremoris*	A	nd	nd	nd	nd
DS68042	*cremoris*	B	nd	nd	nd	nd
DS68041	*cremoris*	A	nd	nd	nd	nd

Profile A: UC509.9 profile with a growth cessation at 3 μg.ml^{-1}; **Profile B:** TP901-1 profile with an equal growth cessation (1.3 and 3 μg.ml^{-1}); **Profile C:** No chemical effect; **Profile D:** Growth cessation at 1.3 μg.ml^{-1}; For the inducible lysates: **nd-** Not determined; # +: presence of phage particles; # -: phage particles not detected; * +: lytic host identified; * -: no lytic host.

Table S1. (cont.)

L. lactis strains	*L. lactis* subsp.	MmC growth profile	Phage particle detection			
			Flow cytometry[#]	Electron microscopy[#]	Plaque assays[#]	Host*
DS68056	*cremoris*	A	nd	nd	nd	nd
DS68057	*cremoris*	A	nd	nd	nd	nd
DS68058	*cremoris*	B	nd	nd	nd	nd
DS68059	*cremoris*	B	nd	nd	nd	nd
DS64886	*cremoris*	A	nd	nd	nd	nd
DS70282	*lactis*	B	+	nd	+	+
DS70372	*cremoris*	B	nd	nd	nd	nd
DS65308	*lactis*	A	nd	nd	nd	nd
DS67635	*cremoris*	B	nd	nd	nd	nd
DS64964	*cremoris*	A	-	nd	nd	nd
DS70385	*lactis*	B	nd	nd	nd	+
DS65028	*cremoris*	B	nd	nd	nd	nd
DS64982	*lactis*	D	+	+	+	+
DS65966	*cremoris*	A	nd	nd	nd	nd
DS64981	*lactis*	B	nd	nd	nd	nd
DS69058	*lactis*	A	nd	nd	nd	nd
DS63624	*lactis*	D	nd	nd	nd	nd
DS63625	*lactis*	D	+	nd	+	+
DS67669	*cremoris*	D	nd	nd	nd	nd
DS63626	*lactis*	B	nd	nd	nd	nd
DS66563	*lactis*	B	-	-	nd	nd
DS67792	*cremoris*	A	nd	nd	nd	nd
DS67740	*cremoris*	B	nd	nd	nd	nd
DS67611	*lactis* biovar diacetylactis	A	nd	nd	nd	nd
DS67634	*lactis* biovar diacetylactis	D	nd	nd	nd	nd
DS63633	*cremoris*	B	+	+	+	+
DS69067	*cremoris*	A	+	+	nd	nd
DS65560	*lactis* biovar diacetylactis	D	nd	nd	nd	nd
DS70248	*lactis* biovar diacetylactis	D	+	+	nd	nd
DS69059	*cremoris*	B	-	nd	nd	nd
DS68504	*cremoris*	A	nd	nd	nd	nd
DS56538	*cremoris*	B	nd	nd	nd	nd
DS56537	*cremoris*	A	nd	nd	nd	nd
DS390	*cremoris*	A	nd	nd	nd	nd
DS0505	*cremoris*	B	nd	nd	nd	nd
DS601	*cremoris*	A	-	-	nd	nd
DS603	*cremoris*	B	nd	nd	nd	nd
DS940	*cremoris*	B	nd	nd	nd	nd

Profile A: UC509.9 profile with a growth cessation at 3 $\mu g.ml^{-1}$; **Profile B:** TP901-1 profile with an equal growth cessation (1.3 and 3 $\mu g.ml^{-1}$); **Profile C:** No chemical effect; **Profile D:** Growth cessation at 1.3 $\mu g.ml^{-1}$; For the inducible lysates: **nd-** Not determined; # +: presence of phage particles; # -: phage particles not detected; * +: lytic host identified; * -: no lytic host.
