# Supplementary material for: Detecting Lactococcus lactis Prophages by Mitomycin C-Mediated Induction Coupled to Flow Cytometry Analysis
Source: Front Microbiol. 2017 Jul 19;8:1343. doi: 10.3389/fmicb.2017.01343 (PMC5515857; doi:10.3389/fmicb.2017.01343)
Supplement: Supplementary file 2 [file Table_2.PDF]

Table S2. Properties of the fifteen inducible lactococcal lysates and their phage-host interaction used in this study.

Inducible features		Phage-host survey		MmC Growth Profile
**Lysogenic strains inducible at 3 μg.ml^{-1} of MmC**	**Inducible prophages**	**SMQ-86**	**3107**	
L. lactis DS68567	56701	+	-	A
L. lactis DS64982	98201	+	-	D
L. lactis DS70282	28201	+	-	B
L. lactis DS68509	50901	+	-	D
L. lactis DS63625	62501	+	-	D
L. lactis DS72183	18301	+	-	D
L. lactis DS68585	58501	+	-	B
L. lactis DS70385	38501	+	-	B
L. lactis DS72160	16001	+	-	A
L. lactis DS72159	15901	+	-	D
L. lactis DS69075	07501	-	+	A
L. lactis DS63633	63301	-	+	B
L. lactis DS68501	50101	-	+	B
L. lactis DS68586	58601	-	+	A
L. lactis DS71865	86501	-	+	A

P**rofile A:** UC509.9 profile with a growth cessation at 3 μg.ml^{-1}; **Profile B:** TP901-1 profile with an equal growth cessation (1.3 and 3 μg.ml^{-1}); **Profile C:** No chemical effect; **Profile D:** Growth cessation at 1.3 μg.ml^{-1}; **+** lytic host identified; **-** no lytic host.
