# Supplementary material for: Detecting Lactococcus lactis Prophages by Mitomycin C-Mediated Induction Coupled to Flow Cytometry Analysis
Source: Front Microbiol. 2017 Jul 19;8:1343. doi: 10.3389/fmicb.2017.01343 (PMC5515857; doi:10.3389/fmicb.2017.01343)
Supplement: Supplementary file 3 [file Image_1.PDF]

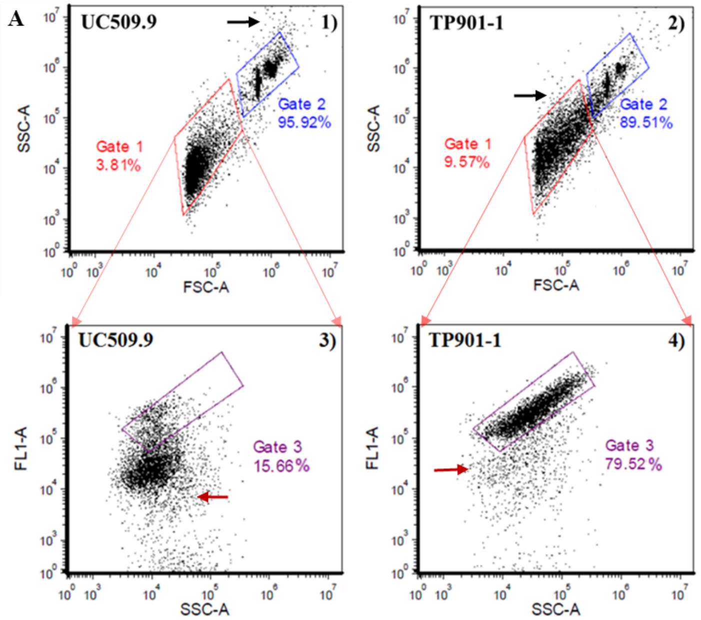

Parameters
Run limits: 5000 events.ml^{-1}
Medium flow rate: 33 µl.min^{-1}
Phage particles detection: SSC (Side Scatter) *versus* FSC (Forward Scatter)
Purpose: Discriminate different particles based on size and complexity in the induced lysate

Analysis
Gate 1- Phage particles population (virions produced by cell lysis)
Gate 2- Microspheres (used as a fluorescence & size reference control)
Gate Outliers- Cell debris and/or background noise (dots outside of each gate; black arrow)

Parameters
Fluorescence detection: FL1 (BP Filter 530/30) *versus* SSC
Purpose: Detect phage DNA particles stained with SYTO-9

Analysis
Gate 3- Phage DNA stained with SYTO-9 in our induced lysate sample (phage population detected in Gate 1)
Gate Outliers- Background noise (dots outside of each gate; red arrow)

Flow cytometry analysis

		% of events (SSC *versus* FSC)			% of fluorescence (FL1 *versus* SSC)	
		**Sample population**	**Beads**	**Cell debris/ noise**	**Phage particles**	**Background noise**
***L. lactis* strains**	**Features**	Gate 1	Gate 2	Outliers of Gates 1 & 2	Gate 3	Outlier of Gate 3
UC509.9	No prophage released (negative control)	6.73 ± 2.07	92.93 ± 2.12	0.34 ± 0.05	16.33 ± 3.76	83.67 ± 3.76
NZ9000 (TP901-1 *erm*)	TP901-1*erm* prophage (positive control)	10.98 ± 1.13	87.97 ± 1.26	1.05 ± 0.15	80.60 ± 2.39	19.39 ± 2.39

Figure S1. Schematic representation of the flow cytometry parameters, analysis and general results for the two *L. lactis* control strains. BD Accuri™ C6 flow cytometer was used for the implementation of the correct parameters to detect and enumerate phage DNA particles stained with SYTO-9 dye. **(A1** and **A3)**: Cytograms of 3 µg.ml^{-1} MmC-treated *L. lactis* UC509.9 (prophage-free lactococcal strain used as negative control); **(A2** and **A4)**: Cytogram of 3 µg.ml^{-1} MmC-treated *L. lactis* NZ9000 TP901-1*erm* (lactococcal strain harbouring the TP901-1 prophage used as a positive control).
